# Personality matters: relationship between personality characteristics, spirituality, demoralization, and perceived quality of life in a sample of end-of-life cancer patients

**DOI:** 10.1007/s00520-021-06363-x

**Published:** 2021-06-24

**Authors:** Ada Ghiggia, Vanni Pierotti, Valentina Tesio, Andrea Bovero

**Affiliations:** 1 Clinical Psychology Unit, AOU Città dela Salute e della Scienza, Corso Bramante 88, 10126 Turin, Italy; 2 Department of Psychology, University of Turin, Via Verdi 10, 10124 Turin, Italy

**Keywords:** Personality, Demoralization, Health-related quality of life, End-of-life, Cancer

## Abstract

**Purpose:**

Personality could be an interesting dimension to explore in end-of-life cancer patients, in order to investigate how personality affects quality of life. Thus, this study aimed to investigate the relationship among personality through the Big Five Inventory (BFI), spirituality, and demoralization and to explore their impact on their quality of life.

**Methods:**

A sample of 210 end-of-life Italian cancer patients were assessed with the BFI, the Demoralization Scale (DS), the Functional Assessment of Chronic Illness Therapy-Spiritual Well-Being (FACIT-SP-12), the Functional Assessment of Cancer Therapy Scale–General Measure (FACT-G), and the Karnofsky performance status.

**Results:**

Correlational analysis highlighted a significantly negative relationship between extraversion and agreeableness traits and all the demoralization dimensions. On the other side, neuroticism trait was significantly and positively correlated with the Demoralization Scale (*p* < 0.01). To understand the impact of these variables on quality of life (FACT-G), we performed a hierarchical multiple regression: in the final model, demoralization remained the strongest contributing factor (β =  − 0.509, *p* < 0.001), followed by neuroticism (β =  − 0.175, *p* < 0.001), spirituality (β = 0.163, *p* = 0.015), and Karnofsky index (β = 0.115, *p* = 0.012).

**Conclusion:**

Our data underlined how both the neuroticism trait and demoralization are correlated with a worst health status in terminal cancer patients, whereas spirituality is a protective factor. The study of personality may allow to better understand the inner patient’s experience and improve communication between patient and healthcare staff in order to build and apply better-tailored psychological treatment.

## Introduction

In recent years, the study of demoralization has arisen as a result of the increasing interest in this syndrome as a relevant clinical dimension in cancer patients [1, 2]. Demoralization is defined as an existential syndrome characterized by feelings of helplessness and hopelessness, a reduced ability to cope, impaired self-esteem, and a loss of purpose and meaning in life [3–5]. Demoralization typically arises from events or situations such as chronic, medical diseases or psychiatric disorders. In such critical moments, the subjective feeling of inability to cope adequately with the event leads the person to perceive him/herself as blocked and unable to react [2, 5, 6]. Many studies on the demoralization syndrome are focused on end-of-life patients and on their experience of disease as a life-threatening or a life-limiting in this regard [2, 7]. Furthermore, it is well documented that this syndrome has a negative impact on patients’ quality of life [8, 9]. Concerning the study of personality, the Big Five Model [10] had an integrative function in the clinical and research field, as evidenced by the increasing number of publications in the last 40 years. The five factors are respectively organized in bipolar dimensions: neuroticism-emotional stability, extraversion-introversion, agreeableness-antagonism, conscientiousness-lack of direction, openness to experience-closed-mindedness. Each factor encompasses more specific traits or personal characteristics: neuroticism represents someone more likely to be apprehensive, anxious, and feel negative emotions; extraversion is associated to energetic excitement, social dominance, and positive feelings; agreeableness represents honesty and regard for others; conscientiousness is linked to planning and self-controlling in decision-making; openness is being curious and open to life’s experiences, thoughts, and variety [11]. Various researches have highlighted the relationship between specific personality traits and diseases like obesity, inflammations, or cardiovascular disease [12, 13]. Moreover, some personality traits are associated with higher mortality risk in both healthy and unhealthy people [12, 14, 15]. In fact, people with specific personality traits are more likely to experience psychopathological symptoms [16, 17].

Given all the above, personality could be an interesting dimension to explore in end-of-life cancer patients, in order to investigate how personality affects quality of life. Since previous studies were conducted into other diseases [18], to our knowledge—besides Chochinov’s study (2006)— no other studies have investigated the link between the different facets of personality with demoralization and the quality of life in terminal cancer patients, increasing the need for explorative studies to understand this relationship better [19]. Moreover, Chochinov et al. (2006) only investigated the neuroticism trait, finding a significant association between this trait and the dying experience.

Given the lack of research on personality characteristics in cancer patients at the end of life and the need to provide targeted psychological interventions, the first aim of our study was to investigate personality through the Big Five Model (through the Big Five Inventory-Italian version) [20] in these patients. Our secondary objective was to explore the relationship between personality characteristics, spirituality, demoralization, and the perceived quality of life at the end of life.

## Methods

### Study design and participants

A descriptive correlational study design was conceived: patients were recruited from January 2018 to March 2019 at the “Città della Salute e della Scienza” Hospital of Turin.

Data collection was conducted by the Clinical Psychology Unit, where a psycho-oncologist of the Unit, specialized in supporting patients at the end of life, provides psychological consultations in the several hospital wards, detecting psychological distress, and providing psychological interventions to end-of-life cancer patients and their caregivers. Inclusion criteria were being 18 years old or more, having been diagnosed with cancer, being hospitalized, and meeting the criteria to access palliative care. According to Piedmont Regional Legislation, the following criteria are required: the presence of an advanced stage of disease (terminal phase) for which every curative treatment is not possible or appropriate and with an unfavorable/poor prognosis, having a presumed life expectancy of 4 months or less, and a Karnofsky performance status [21] of 50 or lower (Piedmont Regional Legislative Decree no. 45/2002 and National Law on Palliative Care and Pain Treatment no. 38/2010). The life expectancy was estimated based on the “surprise question” [22], the Palliative Prognostic Score [23], and the clinical experience of the palliative physician. Exclusion criteria were insufficient knowledge of the Italian language and/or a history of neurological and/or severe psychiatric pathologies.

Patients who agreed to the study and met the inclusion criteria were consecutively enrolled. All the participants provided written informed consent and the present study was approved by the Institutional Ethics Committee (protocol number 0034403, procedure number CS2/1178) and conducted in accordance with the Declaration of Helsinki. The final sample consisted of 210 end-of-life cancer patients. Palliative physicians gathered the sociodemographic and clinical data for each patient. Later on, psychologists interviewed patients at their bedside and administered the Italian validated versions of a set of rating scales.

### Measures

The Big Five Inventory-Italian Version (BFI) [20], based on the Five Factors Theory, was used to examine the individual’s personality traits. The BFI measured the five factors including extraversion, neuroticism, agreeableness, conscientiousness, and openness. It consists of 44 items on a 5-point Likert scale, from 1 (disagree strongly) to 5 (agree strongly). Ubbiali et al. reported internal consistencies (Cronbach’s alphas) ranging from 0.69 to 0.83 along the traits [20].

The Demoralization Scale Italian version (DS-IT) [24], a self-report scale, was used to assess demoralization. It is composed of 24 items on a 5-point Likert scale (from 0 = never to 4 = always). Each item investigates one of the five demoralization subscales (loss of meaning and purpose, dysphoria, disheartenment, helplessness, and sense of failure). A total score of 96 is the maximum score, indicating the index of severity. According to Kissane (2004) [25], a total score of 30 or more indicates high levels of demoralization. Cronbach alpha of the DS-IT was acceptably good (α = 0.80) [24].

The Functional Assessment of Chronic Illness Therapy–Spiritual Well-Being (FACIT-Sp-12) measures spirituality [26]. It is composed of 12 items underlying the traditional religiousness dimension (Faith) and the spiritual (Meaning/Peace) [26]. The wording of the items does not assume a belief in God, so it can also be completed by both atheists and agnostics. The items are on a 5-point Likert scale, ranging from 0 (not at all) to 4 (very much). The total score ranges from zero to 48, with higher scores representing greater levels of spirituality. Internal consistency was adequate with Cronbach’s α ranged from 0.73 to 0.85 [27].

The Functional Assessment of Cancer Therapy Scale–General Measure (FACT-G) is a 27-item scale divided into four primary quality of life domains: physical well-being, social/family well-being, emotional well-being, and functional well-being [28]. Patients provide responses to each question according to a 5-point scale ranging from 0 (not at all) to 4 (very much). The total FACT-G score is the sum of the scores for the four subscales: higher scores indicate higher health-related quality of life (HRQoL). Cronbach alpha of the Italian FACT-G ranged between 0.65 and 0.89 [29].

The KPS is a clinician-rated index of physical-functioning ability that is frequently used in studies of cancer patients [21]. A higher score (range 0–100) means that the patient is able to perform daily activities.

### Statistical analysis

Values of asymmetry and kurtosis between –1 and + 1 were considered acceptable to prove normal univariate distribution of the data. Based on these criteria, the assumption of normality was met for all the variables. Mean (SD) scores and frequencies were used as descriptive analyses. Pearson’s bivariate correlations were used to analyze the relationship between demographic, clinical, and psychological variables. Simple regression analyses were used to analyze the individual association between potentially predictive variables and the HRQoL. A hierarchical multiple linear regression analysis, with stepwise method, was used to investigate whether personality, spirituality, and demoralization were significant contributing factors to the explanation of the HRQoL, using the total score of FACT-G as the outcome variable.

There was linearity, as assessed by partial regression plots and a plot of studentized residuals against the predicted values. Independence of residuals was assessed by a Durbin-Watson statistic of approximately 2. There was homoscedasticity, as assessed by visual inspection of a plot of studentized residuals versus unstandardized predicted values. The tolerance and variance inflation factors were consulted to check for multicollinearity. The P–P plot was consulted to check for normality of the residuals’ distribution. All statistical tests were 2-tailed with a set at 0.05. All the analyses were conducted using the software SPSS Statistics Version 26.0.0 (IBM Corp. Armonk, New York).

## Results

### Demographic, clinical, and psychological characteristics

Table 1 summarizes patient clinical demographics and HRQoL variables.

**Table 1:** Demographic and clinical variables

	*N* = 210
*Sex, N (%)*	
Female	93 (44.3)
Male	117 (55.7)
*Age (y)*	
Mean ± SD	67.8 (11.6)
*Marital status*	
Single	26 (12.4)
Married	129 (61.4)
Divorced	24 (11.4)
Widowed	31 (14.8)
*Educational level, N (%)*	
Basic education (ISCED 0–2)	122 (58.1)
Secondary education (ISCED 3/4)	67 (31.9))
Tertiary education (ISCED 5/6)	21 (10.0)
*Karnofsky performance status index*	
Mean ± SD	39.95 (8.88)
*Cancer site, N (%)*	
Lung cancer	51 (24.4)
Gastrointestinal	34 (16.2)
Genitourinary	29 (13.9)
Hepatic-pancreatic	28 (13.4)
Breast	23 (11.1)
Other site cancers	44 (21)
*FACT-G (Mean* ± *SD)*	
Total score	54.15 (15.04)
Physical well-being	16.33 (4.99)
Social/family well-being	16.03 (5.28)
Emotional well-being	12.43 (5.08)
Functional well-being	9.56 (4.79)

Abbreviations:* HADS *Hospital Anxiety and Depression Scale,* FACT-G *Functional Assessment of Cancer Therapy-General

The final sample consisted of 210 patients with a mean age ± standard deviation (SD) of 67.8 years ± 11.6 (range 33–98). Most of the patients were male (55.7%) and 61.4% of the participants were married.

The KPS mean score was 39.95, highlighting that our sample required special assistance and care because unable to care for themselves. The most frequent types of cancer were lung, hepatic-pancreatic-biliary, breast, and colorectal. Catholicism was the most commonly declared religious affiliation. As regards HRQoL, patients in our sample reported a mean score of 54.2 (± 15.0) for the FACT-G total score (Table 1).

### Psychological variables

Data concerning psychological variables are listed in Table 2.

**Table 2:** Psychological variables, means ± SD

	*N* = 210
Demoralization scale	
Total score	36.2 (14.3)
Loss of meaning and purpose	5.6 (4.4)
Dysphoria	6.0 (3.1)
Disheartenment	12.9 (4.0)
Helplessness	6.3 (3.6)
Sense of failure	5.5 (2.4)
Big Five Inventory (BFI)	
Extraversion	27.8 (6.5)
Agreeableness	34.4 (6.9)
Conscientiousness	36.3 (5.6)
Neuroticism	25.3 (6.3)
Openness to Experience	31.3 (9.1)
FACIT-Sp-12	
Total score	24.3 (8.0)
Meaning/Peace	19.3 (5.6)
Faith	4.9 (3.8)

Abbreviations: *HADS *Hospital Anxiety and Depression Scale, *FACIT-Sp *Functional Assessment of Chronic Illness Therapy-Spiritual Well-Being Scale

Our sample’s mean demoralization total score was 36.2 ± 14.3, regarding the related subscales: loss of meaning and purpose was 5.6 ± 4.4, dysphoria 6.0 ± 3.1, disheartenment 12.9 ± 4.0, helplessness 6.3 ± 3.6, and sense of failure 5.5 ± 2.4.

Regarding BFI scores, the extraversion mean was 27.75 (± 6.55), agreeableness 34.39 (± 6.89), conscientiousness 36.32 (± 5.58), neuroticism 25.26 (± 6.28), and openness 31.25 (± 9.13). Compared to the data reported by Ubbiali [20] from a healthy sample of the Italian population, we found significantly higher values for the extraversion [t(209) = 2.64, *p* < 0.05] and conscientiousness [t(209) = 9.77, *p* < 0.001] traits, while significantly lower levels were detected for the openness trait [t(209) =  − 8.65, *p* < 0.001].

The FACIT-Sp-12 average score was 24.3 ± 8.0, with the two subscales contributing as follows: mean score of 19.3 ± 5.6 for Meaning/Peace, mean score of 4.9 ± 3.8 for Faith.

### Correlational analysis: personality, demoralization, and spirituality dimensions

Table 3 shows the correlations between the BFI traits, demoralization, and the spirituality dimension. The main findings were for extraversion and agreeableness BFI traits, which showed a significantly negative correlation with all the demoralization dimensions. In line with these results, the neuroticism trait was significantly and positively correlated with all the subscales of the demoralization scale (DS-IT) (*p* < 0.01). On the other hand, the conscientiousness and openness to experience traits showed a negative correlation with all the demoralization dimensions, albeit with a low correlation.

**Table 3:** Pearson’s correlations among psychological variables

	BFI						
	Extraversion	Agreeableness	Conscientiousness	Neuroticism	Openness to experience	Age	Karnofsky
Age	− 0.029	0.054	0.062	0.115	− 0.169*	-	-
Karnofsky	− 0.151*	− 0.198**	− 0.035	− 0.151*	0.171*	-	-
DS-IT	− 0.395**	− 0.235**	− 0.164*	0.475**	− 0.233**	0.080	− 0.197*
DS-IT loss of meaning	− 0.415**	− 0.218**	− 0.145*	0.392**	− 0.270**	0.175*	− 0.188*
DS-IT dysphoria	− 0.269**	− 0.218**	− 0.145*	0.392**	− 0.270**	− 0.05	− 0.103
DS-IT disheartenment	− 0.266**	− 0.136*	− 0.067	0.432**	− 0.093	0.038	− 0.170*
DS-IT helplessness	− 0.334**	− 0.173*	− 0.105	0.475**	− 0.169*	0.072	− 0.176*
DS-IT sense of failure	− 0.478**	− 0.109	− 0.313**	0.341**	− 0.263**	0.059	− 0.147*
FACIT-Sp total score	0.368**	0.257**	0.139*	− 0.341**	0.216**	− 0.027	0.135
FACIT-Meaning/Peace	0.471**	0.203**	0.183**	− 0.447**	0.308**	− 0.101	0.188*
FACIT-Faith	0.103	0.226**	0.021	− 0.060	0.018	0.094	0.008

Abbreviations: *BFI* Big Five Inventory; *FACIT-Sp* Functional Assessment of Chronic Illness Therapy-Spiritual Well-Being Scale; *DS-IT* Demoralization Scale Italian version

*p value < 0.05; **p value ≤ *0.01*

As regards spirituality, we found that the extraversion and openness to experience traits show a significant positive correlation with the total score and the Meaning/Peace scale of FACIT-Sp-12 (*p* < 0.01). However, neuroticism was negatively correlated with spirituality (FACIT-Sp total score and Meaning/Peace subscale; *p* < 0.01).

Furthermore, negative correlations were found between age and the openness to experience trait (*p* < 0.05) and between KPS and extraversion (*p* < 0.05), KPS and agreeableness (*p* < 0.01), and KPS and neuroticism (*p* < 0.05).

### Relationship among psychological and personality variables and the HRQoL

The results of the simple regression analyses on the HRQoL are listed in Table 4. The demoralization scale showed a strong significant and negative association with the HRQoL (FACT-G total score; β =  − 0.736, *p* ≤ 0.01), whereas spirituality instead was significantly and positively associated with the FACT-G total score (*p* ≤ 0.01). All personality traits were significantly associated with the FACT-G total score (*p* < 0.01).

**Table 4:** Simple associations among sociodemographic characteristics, psychological and personality variables, and the HRQoL

	FACT-G total score		
	*β*	*t*	*p value*
Age	− 0.073	− 1.06	0.290
Karnofsky	0.264	3.95	< *0.001*
DS-IT total score	− 0.736	− 15.67	< *0.001*
FACIT-Sp total score	0.616	11.29	< *0.001*
BFI extraversion	0.342	5.24	< *0.001*
BFI agreeableness	0.211	3.12	*0.002*
BFI conscientiousness	0.194	2.85	*0.005*
BFI neuroticism	− 0.489	− 8.09	< *0.001*
BFI openness to experience	0.199	2.93	*0.004*

Abbreviations: *BFI* Big Five Inventory; *FACT-G* Functional Assessment of Cancer Therapy-General; *DS-IT* Demoralization Scale Italian version; *FACIT-Sp* Functional Assessment of Chronic Illness Therapy-Spiritual Well-Being Scale

Regarding the index of physical-functioning ability, a negative association (*r* =  − 0.632; *p* < 0.01) was found between Karnofsky and the FACT-G total score.

### Regression analyses

A hierarchical multiple regression analysis, with stepwise method, was run to examine the additive ability of personality traits, in addition to demoralization and spirituality, to predict the health-related quality of life. Based on the literature and on the results of the previous analyses, the clinical variable (KPS) was introduced in the first block; the psychological variables (demoralization and spirituality) in the second block; and the exploratory variables (i.e., the personality traits) in the third block. The regression model is reported in Table 5.

**Table 5:** Hierarchical multiple regression with quality of life (FACT-G) as dependent variable

Predictor	*B*	*β*	*t*	*R*^*2*^	*Adj-R*^*2*^	*ΔR*^*2*^	*F*
*Step 1*				0.07	0.07	0.07**	15.63**
Karnofsky index	0.447	0.264	3.95**				
*Step 2*				0.56	0.55	0.49**	129.7**
Karnofsky index	0.210	0.124	2.62*				
DS-IT	− 0.751	− 0.711	− 15.06**				
*Step 3*				0.57	0.56	0.01*	90.07**
Karnofsky index	0.213	0.126	2.69*				
DS-IT	− 0.626	− 0.593	− 8.57**				
FACIT-SP total score	0.299	0.159	2.32*				
*Step 4*				0.59	0.58	0.02**	74.04**
Karnofsky index	0.195	0.115	2.53*				
DS-IT	− 0.537	− 0.509	− 7.09**				
FACIT-SP total score	0.307	0.163	2.44*				
BFI neuroticism	− 0.419	− 0.175	− 3.43**				

*p value < 0.05; ** p value < 0.001

Abbreviations: FACIT-Sp Functional Assessment of Chronic Illness Therapy-Spiritual Well-Being Scale; DS-IT Demoralization Scale Italian version; BFI Big Five Inventory

Regarding the psychological variables, the first to be introduced in the model was the demoralization (step 2), which lead to the highest increase in the proportion of variance explained (Δ*R*^2^ = 0.49, *F*(1207) = 226.8, *p* < 0.001). When also spirituality entered the model (step 3), demoralization remained the strongest contributing factor (β =  − 0.593, *t*(206) =  − 8.57, p < 0.001). The first, and only, personality trait showing a significant improvement in the model predictive ability was the neuroticism factor of the BFI (step 4). The neuroticism trait improved the model, significantly and negatively impacting on the HRQoL, but did not substantially change the weight of the psychological variables suggesting that all these variables play an independent contributing role. No other personality traits further improved the model, and thus they were not included in the regression. The final model (step 4) predicts 58% of the variance of the HRQoL (*F*(4, 205) = 74.04, *p* < 0.001). Demoralization remained the strongest contributing factor (β =  − 0.509, *t*(205) =  − 7.09, *p* < 0.001), followed by neuroticism (β =  − 0.175, *t*(205) =  − 3.43, *p* < 0.001), spirituality (β = 0.163, *t*(205) = 2.44, *p* = 0.015), and Karnofsky index (β = 0.115, *t*(205) = 2.53, *p* = 0.012).

## Discussion

The present study aimed to explore the relationship between the facets of personality and demoralization and spirituality dimensions and their impact on a sample of end-of-life cancer patients’ quality of life. To the best of our knowledge, no study to date has focused on the different impacts that these dimensions have on health-related quality of life in terminal cancer patients.

Regarding the personality trait, following the Big Five construct, our sample showed some significant differences in personality compared to the normative data on an Italian sample [20], with specific regard to the extraversion, conscientiousness, and openness traits. These results may depend on multiple factors: our sample was composed of end-of-life cancer patients, older than those in the study by Ubbiali et al. [20]. In this regard, some studies have suggested that the conscientiousness trait slightly increases over the years, and on the other hand, the openness to experience trait tends to decrease [30]. Thus, more conscientious individuals perceived themselves as being more capable at managing difficulties at the end of life, preserving their decision-making ability and being able to maintain a certain degree of personal agency and preserve their sense of dignity [31, 32]. Furthermore, it is well documented that a traumatic event, such as a diagnosis of cancer or a terminal illness, has a huge impact on patients’ personality [33]. Unlike the existing literature about personality traits in the end of life, in which increasing functional limitation decreases the extroversion trait, we found that levels of extraversion were higher in late life. Therefore, those who felt less lonely because they were engaged in social relationships were more extraverted and enjoyed a better quality of life [34]. These findings supported the close relationship between the development of personality and the social relationships in the end of life, underlining the interdependence and the reciprocal influence between dying patients and caregivers.

A further step of our study was to explore the relationship between personality traits and demoralization. We found significantly negative correlations among the extraversion, agreeableness, conscientiousness and openness traits, and demoralization scores, suggesting that scored higher in these dimensions tended to experience less this existential distress.

And, as expected, the extraversion, agreeableness, conscientiousness, and openness to experience traits showed also significant and positive association with the spirituality dimension, particularly with the spiritual dimension (Meaning/Peace subscale of the FACIT-Sp-12). These data suggested that some personality characteristics can be considered protectors’ factors on well-being, predisposing individual to remain involve with spiritual practice and to receive the benefit of it. On the other hand, neuroticism could be a risk factor for affective disorder: patients with low level of religiosity and spirituality tended to report elevated levels of psychopathology. In fact, spirituality may help reduce negative emotionality and increase distress tolerance or facilitate adaptive coping strategies [35].

Using the literature framework, we can try to understand this evidence by bringing together the main characteristic of each trait and our results. For example, it has been observed that extrovert individuals experienced a stronger responsiveness to positive emotions and higher motivation to pursue them [36, 37]. We may assume that in our sample this personality substratum towards positive emotions can facilitate experiencing such positive spiritual meaning and peace and a more general well-being; and, on the other hand, it could be a protective variable against loss of meaning and sense of failure.

Similar to the extroversion facet, our data showed that patients who are honest and respectful towards others (agreeableness) seem to exhibit better general well-being. In fact, people with a high score on agreeableness can be described as empathetic [38]: relatives see them as trusting, cooperative, motivated, and sensitive, so it seems unlikely that these people can experience a condition of demoralization. This seems to be suggested in the significantly negative correlation between this trait and demoralization, reflecting how certain aspects of the personality are a protective factor against mood alteration and loss of meaning. Furthermore, it is interesting that the agreeableness trait is the only one associated with Faith (FACIT-Sp-12), suggesting that those individuals tend to view the future in a more confident and hopeful way [39].

Regarding openness to experience, this trait also appears to be a protective factor against the demoralization syndrome in end-of-life patients. These individuals are generally open, with a vivid imagination and fantasy life, particularly responsive to music, art, or emotions, reporting more unusual and spiritual experiences [40]. This may reinforce spiritual beliefs and seems to be expressed by the association between this trait and the FACIT-Sp-12, particularly with the Meaning/Peace dimension. Moreover, a similar effect due to this inner relationship with diversity and curiosity can be found in the negative relationship between openness and loss of meaning (DS). Thus, open people are more curious, creative, and motivated to explore the world and embrace its possibilities. These people therefore have a different attitude when coping with problems and dealing with life’s challenges.

Regarding neuroticism, our findings confirmed previous studies: this trait is particularly associated with maladaptive coping strategies and a worse outcome in end-of-life patients [19, 31]. The neuroticism trait represents the inner responsiveness to negative feelings and the way that people react to them [38]. Chochinov already demonstrated that neuroticism has a large impact on HRQoL on mood and also on the sense of dignity [19]. In addition, our findings point out that with hopelessness (DS), all the demoralization subscales are closely associated with the neuroticism trait. Moreover, as expected, patients with high scores in this trait seem to experience less spiritual peace and meaning (FACIT-Sp-12), being less protected from existential discomfort. They are naturally susceptible to negative feelings and this aspect is amplified during the end-of-life phase, where people often have to cope with these emotions, from small daily loss to grief [38]. Those who appear most apprehensive and anxious as they approach death have more negative thoughts, tend to complain more, and have more difficulty in dealing with the end of life and grief than those who are able to manage their emotions and impulses.

Regarding quality of life, our data allow us to underline how both the neuroticism trait and demoralization are correlated with a negative index of health status. Indeed, in the hierarchical multiple regression analysis performed on the HRQoL, neuroticism significantly contributed to the model even when controlling for the presence of clinical and psychological variables, as demoralization and spirituality. Karnofsky index, demoralization, and the neuroticism trait were all significant predictors of a worse HRQoL. On the contrary, spirituality was a protective factor, showing a significantly positive impact on the perceived quality of life. No other personality traits seem to play a key role in this model, and thus contributing to the explanation of the HRQoL in end-of-life cancer patients, with a final model explaining a high percentage of the variance (around 60%).

Both neuroticism trait and demoralization seem to cause the worst outcomes in terminal cancer patients, specifically with regard to health-related quality of life [31, 41, 42]. It is possible that high levels of neuroticism mediate the end-of-life distress (symptoms, concerns) influencing how dying patients cope with this experience and that, in the same way, spirituality provides a protective element in this delicate phase of life.

It could be argued that the persistent incapacity of coping with end-of-life distress can arise the level of demoralization, thereby weakening the patient’s quality of life. Thus, it seems crucial to deep-in how the individual faces the difficulties and emotions that accompany him/her at the end-of-life cycle [43].

The changes and difficulties faced at the end of life in both physical and mindset functioning could lead to an increase in neuroticism, augmenting feelings of helplessness and psychological distress related to the severe mental and physical limitations. From a clinical perspective, the current data could have important implications. Personality could play a role in the development of the demoralization syndrome and could influence patients’ functioning and adaptation to their life situation.

Following these considerations, it is important to have information on personality to better understand the inner patient’s experience and improve communication between patient and healthcare staff in order to build and apply better-tailored psychological treatment.

## Limitations

The study has some limitations, although the Five Personality Factor model may be a cross-culturally solid and transversal construct of inner characteristics. It would be useful to integrate the research with different theoretical frames related to the study of personality in terminal cancer patients. Furthermore, longitudinal studies will also be needed to better understand the resonance of a terminal diagnosis on personality characteristics.

We would expect that an inclusion of social behavioral and interpersonal perception in the near future of the end-of-life studies could show which type of interactions (e.g., with spouse, healthcare staff, friends) are able to impact on the development of personality traits. Further research is needed to understand how increased functional limitations (physical, cognitive, and psychological disabilities) and the mortality process affect personality near death.

## Conclusion

The current study contributes to the existing considerations of personality traits at the end of life, analyzing the full five-factor personality model (neuroticism, extraversion, openness to experience, agreeableness, and conscientiousness). Our results underlined how both the neuroticism trait and demoralization are predictive of a worst health status in terminal cancer patients, whereas spirituality seems to be a protective factor. The possibility to study the personality characteristics of dying patients would allow clinical psychologists to better investigate the approach and coping strategies that the patient adopts to deal with end-of-life challenges and related concerns.

## Data Availability

Data are available from the corresponding author upon reasonable request.

## References

[1] Fang CK, Chang MC, Chen PJ, Lin CC, Chen GS, Lin J, Hsieh RK, Chang YF, Chen HW, Wu CL, Lin KC, Chiu YJ, Li YC (2014). A correlational study of suicidal ideation with psychological distress, depression, and demoralization in patients with cancer. Support Care Cancer.

[2] Bovero A, Botto R, Adriano B, Opezzo M, Tesio V, Torta R (2019). Exploring demoralization in end-of-life cancer patients: Prevalence, latent dimensions, and associations with other psychosocial variables. Palliat Support Care.

[3] Clarke DM, Kissane DW (2002). Demoralization: its phenomenology and importance. Aust N Z J Psychiatry.

[4] Norris L, Pratt-Chapman M, Noblick JA, Cowens-Alvarado R (2011). Distress, demoralization, and depression in cancer survivorship. Psychiatr Ann.

[5] Robinson S, Kissane DW, Brooker J, Burney S (2015). A systematic review of the demoralization syndrome in individuals with progressive disease and cancer: a decade of research. J Pain Symptom Manage.

[6] De Weert GH, Markus W, Kissane DW, De Jong CAJ (2017). Demoralization in Patients With Substance Use and Co-Occurring Psychiatric Disorders. J Dual Diagn.

[7] Robinson S, Kissane DW, Brooker J, Burney S (2016). A Review of the Construct of Demoralization: History, Definitions, and Future Directions for Palliative Care. Am J Hosp Palliat Care.

[8] Robinson S, Kissane DW, Brooker J, Hempton C, Burney S (2017). The Relationship Between Poor Quality of Life and Desire to Hasten Death: A Multiple Mediation Model Examining the Contributions of Depression, Demoralization, Loss of Control, and Low Self-worth. J Pain Symptom Manage.

[9] Nanni MG, Caruso R, Travado L, Ventura C, Palma A, Berardi AM, Meggiolaro E, Ruffilli F, Martins C, Kissane D, Grassi L (2018). Relationship of demoralization with anxiety, depression, and quality of life: A Southern European study of Italian and Portuguese cancer patients. Psychooncology.

[10] John OP, Naumann LP, Soto CJ (2008). Paradigm shift to the integrative Big Five trait taxonomy: History, measurement, and conceptual issues. Handbook of Personality: Theory and Research.

[11] Costa PT, McCrae RR (2008) The revised NEO personality inventory (NEO-PI-R). In The SAGE Handbook of Personality Theory and Assessment: Volume 2 - Personality Measurement and Testing (179–198). SAGE Publications Inc. 10.4135/9781849200479.n9

[12] Kupper N, Denollet J (2018). Type D Personality as a Risk Factor in Coronary Heart Disease: a Review of Current Evidence. Curr Cardiol Rep.

[13] Wagner EN, Ajdacic-Gross V, Strippoli MF, Gholam-Rezaee M, Glaus J, Vandeleur C, Castelao E, Vollenweider P, Preisig M, von Känel R (2019). Associations of Personality Traits With Chronic Low-Grade Inflammation in a Swiss Community Sample. Front Psychiatry.

[14] Friedman HS, Kern ML, Reynolds CA (2010). Personality and health, subjective well-being, and longevity. J Pers.

[15] McCann SJH (2014). Higher Resident Neuroticism Is Specifically Associated With Elevated State Cancer and Heart Disease Mortality Rates in the United States. SAGE Open.

[16] Widiger TA, Trull TJ (1992). Personality and psychopathology: an application of the five-factor model. J Pers.

[17] Soldz S, Vaillant GE (1999). The Big Five Personality Traits and the Life Course: A 45-Year Longitudinal Study. J Res Pers.

[18] Rassart J, Luyckx K, Verdyck L, Mijnster T, Mark RE (2020). Personality functioning in adults with refractory epilepsy and community adults: Implications for health-related quality of life. Epilepsy Res.

[19] Chochinov HM, Kristjanson LJ, Hack TF, Hassard T, McClement S, Harlos M (2006). Personality, neuroticism, and coping towards the end of life. J Pain Symptom Manage.

[20] Ubbiali A, Chiorri C, Hampton P (2013) Italian Big Five Inventory. Psychometric properties of the Italian adaptation of the Big Five Inventory (BFI). BPA-Applied Psychology Bulletin (Bollettino di Psicologia Applicata), 59(266)

[21] Schag CC, Heinrich RL, Ganz PA (1984). Karnofsky performance status revisited: reliability, validity, and guidelines. J Clin Oncol.

[22] Moss AH, Lunney JR, Culp S, Auber M, Kurian S, Rogers J, Dower J, Abraham J (2010). Prognostic significance of the "surprise" question in cancer patients. J Palliat Med.

[23] Maltoni M, Nanni O, Pirovano M, Scarpi E, Indelli M, Martini C, Monti M, Arnoldi E, Piva L, Ravaioli A, Cruciani G, Labianca R, Amadori D (1999). Successful validation of the palliative prognostic score in terminally ill cancer patients. Italian Multicenter Study Group on Palliative Care. J Pain Symptom Manage.

[24] Costantini A, Picardi A, Brunetti S, Trabucchi G, Bersani FS, Minichino A, Marchetti P (2013). La versione italiana della Demoralization Scale: uno studio di validazione [Italian version of Demoralization Scale: a validation study]. Riv Psichiatr.

[25] Kissane DW, Wein S, Love A, Lee XQ, Kee PL, Clarke DM (2004). The Demoralization Scale: a report of its development and preliminary validation. J Palliat Care.

[26] Peterman AH, Fitchett G, Brady MJ, Hernandez L, Cella D (2002). Measuring spiritual well-being in people with cancer: the functional assessment of chronic illness therapy–Spiritual Well-being Scale (FACIT-Sp). Ann Behav Med.

[27] Rabitti E, Cavuto S, Iani L, Ottonelli S, De Vincenzo F, Costantini M (2020). The assessment of spiritual well-being in cancer patients with advanced disease: which are its meaningful dimensions?. BMC Palliat Care.

[28] Cella DF, Tulsky DS, Gray G, Sarafian B, Linn E, Bonomi A, Silberman M, Yellen SB, Winicour P, Brannon J (1993). The functional assessment of cancer therapy scale: development and validation of the general measure. J Clin Oncol.

[29] Bonomi AE, Cella DF, Hahn EA, Bjordal K, Sperner-Unterweger B, Gangeri L, Bergman B, Willems-Groot J, Hanquet P, Zittoun R (1996). Multilingual translation of the functional assessment of cancer therapy (FACT) quality of life measurement system. Qual Life Res.

[30] Costa PT, Weiss A, Duberstein PR, Friedman B, Siegler IC (2014). Personality facets and all-cause mortality among Medicare patients aged 66 to 102 years: a follow-on study of Weiss and Costa (2005). Psychosom Med.

[31] Bovero A, Cotardo F, Pierotti V, Gottardo F, Botto R, Opezzo M, Geminiani GC (2021). Personality traits and sense of dignity in end-of-life cancer patients: a cross-sectional study. Am J Hosp Palliat Care.

[32] Vehling S, Kissane DW (2018). Existential distress in cancer: alleviating suffering from fundamental loss and change. Psychooncology.

[33] Specht J, Egloff B, Schmukle SC (2011). Stability and change of personality across the life course: the impact of age and major life events on mean-level and rank-order stability of the Big Five. J Pers Soc Psychol.

[34] Berg AI, Johansson B (2014). Personality change in the oldest-old: is it a matter of compromised health and functioning?. J Pers.

[35] McCullough ME, Willoughby BL (2009). Religion, self-regulation, and self-control: associations, explanations, and implications. Psychol Bull.

[36] Larsen RJ, Ketelaar T (1991). Personality and susceptibility to positive and negative emotional states. J Pers Soc Psychol.

[37] Gross JJ, Sutton SK, Ketelaar T (1998). Relations between affect and personality: support for the affect-level and affective-reactivity views. Pers Soc Psychol Bull.

[38] Nettle D (2007) Personality: What makes you the way you are. Oxford University Press

[39] Chaar EA, Hallit S, Hajj A, Aaraj R, Kattan J, Jabbour H, Khabbaz LR (2018). Evaluating the impact of spirituality on the quality of life, anxiety, and depression among patients with cancer: an observational transversal study. Support Care Cancer.

[40] Kandler C, Riemann R, Angleitner A, Spinath FM, Borkenau P, Penke L (2016). The nature of creativity: the roles of genetic factors, personality traits, cognitive abilities, and environmental sources. J Pers Soc Psychol.

[41] Townsend K (2018). Demoralisation in palliative care. Lancet Oncol.

[42] Vanbutsele G, Van Belle S, Surmont V, De Laat M, Colman R, Eecloo K, Naert E, De Man M, Geboes K, Deliens L, Pardon K (2020). The effect of early and systematic integration of palliative care in oncology on quality of life and health care use near the end of life: a randomised controlled trial. Eur J Cancer.

[43] Faguet GB (2016). Quality end-of-Life cancer care: An overdue imperative. Crit Rev Oncol Hematol.

